# The Ethics of Translational Audiology

**DOI:** 10.3390/audiolres12030028

**Published:** 2022-05-13

**Authors:** Aleksandra Bendowska, Roksana Malak, Agnieszka Zok, Ewa Baum

**Affiliations:** 1 Department of Social Sciences and the Humanities, Poznan University of Medical Sciences, 60-806 Poznan, Poland; ebaum@ump.edu.pl; 2 Department and Clinic of Rheumatology, Rehabilitation and Internal Diseases, Poznan University of Medical Sciences, 61-545 Poznan, Poland; rmalak@ump.edu.pl; 3 Division of Philosophy of Medicine and Bioethics, Poznan University of Medical Sciences, 60-806 Poznan, Poland; agzok@ump.edu.pl

**Keywords:** ethics, audiology, deafness, translational research

## Abstract

Translational research moves promising primary research results from the laboratory to practical application. The transition from basic science to clinical research and from clinical research to routine healthcare applications presents many challenges, including ethical. This paper addresses issues in the ethics of translational audiology and discusses the ethical principles that should guide research involving people with hearing loss. Four major ethical principles are defined and explained, which are as follows: beneficence, nonmaleficence, autonomy, and justice. In addition, the authors discuss issues of discrimination and equal access to medical services among people with hearing loss. Despite audiology’s broad field of interest, which includes evaluation and treatment of auditory disorders (e.g., deafness, tinnitus, misophonia, or hyperacusis) and balance disorders, this study focuses primarily on deafness and its therapies.

## 1. Introduction

Audiology is a large branch of medicine concerned with disorders such as hearing loss, hyperacusis (distorted loudness perception causing several noises unbearable and painfully loud to the affected person [1]), tinnitus (the perception of sounds without actual acoustic stimuli [2]), or misophonia (an emotional reaction to sounds [3]). Another branch of audiology deals with vestibular dysfunctions originating from the inner ear [4,5,6].

Hearing loss may have congenital or acquired origins [7], and several therapeutic options were developed to treat the affected patients. These options depend on the site of the pathological changes (outer, middle, inner ear; brainstem or central auditory system) and on the degree of the hearing loss (mild, moderate, or severe) [8]. The National Health Service should support people who are hard of hearing and help to early detect hearing impairment [9]. This article focuses on deafness as a disease model and uses that model to discuss the ethical principles in translational audiology.

Translational research is a relatively new but rapidly growing field in biomedical research that aims to transfer scientific discoveries to clinical practice and to analyze clinical observations in the laboratory. Translational research is also referred to as “from bench to bedside”. The reverse relationship—“from bedside to table”—is also essential [10]. Many discoveries are made in the clinic by observing a patient’s response to treatment. For example, in the clinic, one can observe the correlation of a particular substance, whose biological role we do not understand, with the patient’s clinical state. The researchers try to understand its meaning by going back to the laboratory. One can use the knowledge gained to develop a more effective clinical treatment [11]. The core of translational research is synthesizing information obtained from multiple research sources. Such an approach improves our understanding of human physiology, broadens our knowledge of diseases, and accelerates the discovery and testing of new diagnostic and therapeutic methods. The goal of translational research is to increase the quality and efficiency of medical care for patients.

Ethical perspectives recognize that groups of people who are particularly vulnerable to dignity violation and exploitation are marginalized and socially stigmatized, for example, because of illness, substance abuse, poverty, homelessness, ethnicity or cultural identification, or incapacity. However, it is essential to remember that many people who are medically defined as people with disabilities are fully functional. Discrimination can occur due to limiting access to goods or treating people worse by trying to help them against their needs. Considering deafness in this aspect turns out to be extremely important.

Deafness, due to the perceptions and living reality of the person, can be considered in the following two ways: medical and cultural. [12]. In a medical context, deafness refers to hearing loss and, in that context, is written with a lowercase. In a cultural context, to refer to those who primarily communicate through sign language, regardless of hearing ability, often written with a capital letter as Deaf and referred to as “big D” in speech and sign language [13]. Moral dilemmas arising from the multidimensionality of the concept of Deafness seem extremely difficult to resolve; therefore, it is necessary to know both the history of Deafness, related diseases, and existing codes and ethical norms.

## 2. Deafness in Translational Studies

When conducting translational research with Deaf people, one must also consider the reluctance of this community to participate in genetic testing. This reluctance may stem from the apparent medicalization of deafness, as pointed out by Harlan Lane [14]. This is related to the history of the Deaf, in which genetic engineering methods and medical procedures were used to eliminate deafness. Forced sterilization of Deaf people and conducting genetic testing for congenital deafness are procedures that were used between 1880 and 1950 in the U.S., Britain, and Germany, among others, as elements of the eugenics movement, which aimed to “reduce social burdens” and improve the condition of the human species [15]. To this day, genetic research on deafness in some Deaf communities is met with criticism. In addition, the Deaf community’s reluctance and distrust of scientists are compounded by the definition of deafness in biomedical science as a disability, defect and disease. For some deaf communities, deafness might be a unique feature. Despite all the personal and technical aids and the fact that hearing impairment implies functional limitations in social life, the persons are able to organize their social and cultural life, which is why some deaf people do not opt for preventive screenings or treatments to improve their hearing or prevent hearing loss. On the other hand, however, it should also be emphasized that genetic testing can benefit the Deaf community by identifying an increased risk of conditions associated with some types of congenital deafness, such as retinal pigmentary degeneration (e.g., Usher syndrome), facial dysmorphia (e.g., Treacher-Collins syndrome), long QT syndrome (e.g., Jervell andLange-Nielsen syndrome) and renal dysfunction (e.g., Alport syndrome) [16]. Thus, when including a person with deafness in translational research, one should avoid misunderstandings by taking into account the welfare of the research participant. Moreover, the participant’s safety should be taken into account by assessing the participant’s background, mode of communication, and cultural identification.

This is possible by using the Roman Jakobson’s model of the communicative functions of language. Jakobson’s diagram includes the following six elements: sender, receiver, message, context, code and contact. The sender sends a message to the receiver. This message has a referential context that the receiver can understand. The context includes all the circumstances accompanying a message that have a key impact on it, e.g., time and place, audience, cultural origin and history. It refers to objects, facts and phenomena from the extra-linguistic world. It is also necessary that the sender and receiver share a common code, by which the first one modifies the message and the second, decodes it. In the case of deaf people, sign language may be a common code with hearing people. The last element is contact, which for Jakobson is a physical channel and a psychological connection between the sender and receiver. This allows to both establish and maintain communication.

**Figure 1:**
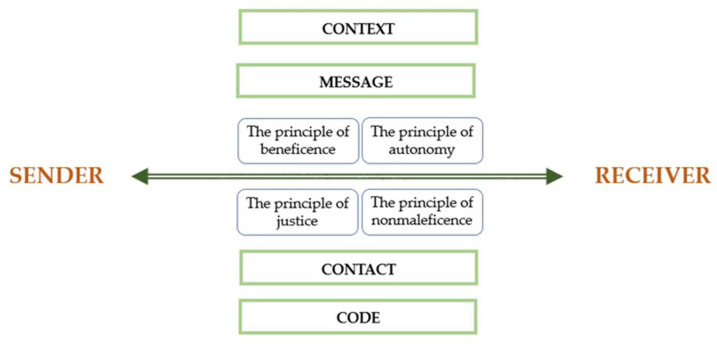
Roman Jakobson’s model of the communicative functions of language with ethical principles in translational research in audiology. Source: own elaboration based on Jakobson, R. Linguistics and Poetics. In Style in Language; Sebeok, T., Ed.; M.I.T. Press: Cambridge, USA, 1960; pp. 350–377.

In the case of translational research in audiology, we recommend enriching Jakobson’s model with ethical principles (Figure 1).

Ethical rules related to the safety of research participants, minimizing harm and abuse of subjects, appeared only in the early 20th century. In 1931, the German Reich published the first ever national set of rules defining the principles for implementing experiments on humans. The document introduced the division of experiments into therapeutic and non-therapeutic. It also emphasized the need to respect the principle of nonmaleficence and beneficence. The regulations included the principle of respecting the autonomy of research participants and obtaining the participant’s (or his representative) consent [17]. Unfortunately, this document did not stop Nazi medics from using barbaric experimental methods on concentration camp prisoners.

Nevertheless, the events during World War II contributed to the establishment of bioethics as a science and the development of critical ethical codes. After the Nuremberg Trials, the most significant war criminals were tried, and doctors were directly responsible for unethical experiments. The Nuremberg Code was formulated—10 principles that researchers should follow during experiments on humans. The Code was the first international attempt to define standards of ethics in scientific research and the first document that unambiguously articulated the requirement of obtaining informed consent of the individual to undertake. In addition, in response to the wartime experience, the World Medical Association developed a modern international version of the Hippocratic Oath, the Geneva Declaration [18], and later, in 1964, one of the most critical sets of ethical principles for medical research, the Helsinki Declaration, putting the welfare of research participants first [19]. This principle was not followed during the scandalous observational study of untreated syphilis, the Tuskegee Syphilis Study, which was conducted between 1932 and 1972. The study had racist overtones, as only black, poor, and poorly educated residents of rural Macon County, Alabama, participated. Effective treatment for syphilis was discovered in the early 1950s, yet the study participants were not given treatment, and observation continued [20]. The Tuskegee Syphilis Study disclosure scandal contributed to the United States Congress establishing the National Commission for the Protection of Participants in Biomedical and Behavioral Research. In 1979, this commission published the Belmont Report. This document pointed out the fundamental differences between research activities directed toward the advancement of scientific knowledge and clinical practice focused on the welfare of the individual patient and identified the following three basic principles for the conduct of research involving human participants in medicine: the principle of respect for the person, the principle of beneficence, and the principle of justice. Over time, these principles have come to be recognized as the fundamental principles of researcher ethics [21].

According to the Belmont Report, respect for the person is expressed in the belief that he or she should be treated as an autonomous agent of action and that persons with diminished autonomy are entitled to protection. Thus, the principle of respect refers to respecting the autonomy of the research participant. Another principle, charity, is understood in the document as an obligation, rather than as an act of kindness or mercy. Two general rules are formulated that provide complementary accounts of acts of kindness thus understood, which are as follows: do no harm, maximize possible benefits, and minimize potential harm. The principle of justice refers to the ethical obligation to treat all people in a manner consistent with what is considered morally proper and speaks to the fair distribution of burdens and benefits that accompany participation to all community members [22].

The four ethical principles, beneficence, nonmaleficence, autonomy, and justice, form the basis for most decision-making in both the research setting and in clinical practice. The following section presents their application both in conducting research in the field of translational audiology and in therapy of people with hearing impairment.

## 3. Basic Principles of Medical Ethics

### 3.1. The Principles of Beneficence and Nonmaleficence

The principle of beneficence is the obligation of the physician to act for the benefit of the patient, prevent harm, remove conditions that will cause harm and help persons with disabilities. It is worth emphasizing that, in distinction to nonmaleficence, the language here is one of the positive requirements. The principle calls for not just avoiding harm, but also to benefit patients and to promote their welfare.

The practical application of nonmaleficence is for the physician to analyze the benefits and losses of the proposed interventions and therapies. The physician is obliged by this principle to avoid those that are inappropriate and chose the best procedure for the patient.

The application of the principle of beneficence in clinical trials refers to a researcher’s obligation to maximize the benefits of the research and minimize the risk of harm to its participants. Therefore, this principle also includes the principle of nonmaleficence, which sets specific limits for the activities of researchers. It prohibits taking any action that may cause intentional evil or harm to the participants of the research, whereby both physical wrong or harm, i.e., one concerning the state of somatic health, as well as emotional and financial harm, must be included.

A key measure ensuring compliance with the principle of beneficence in scientific research is the analysis and assessment of the risk-benefit ratio of a research participant. Research should not impose risks and burdens disproportionate to the expected benefits of participating in it. In the case of experimental research involving sick persons, which gives participants a chance to obtain a direct diagnostic, therapeutic or prophylactic benefit, the benefit-risk balance of the new intervention cannot be assumed to be less favorable than that of the existing best-proven interventions. In the case of scientific research that does not provide direct diagnostic, therapeutic or prophylactic benefits to participants, the accompanying risk should be estimated proportionally to the scientific value intended to be obtained in connection with the implementation of the study. Such tests are allowed only if they involve risks and burdens that are not greater than acceptable [23].

It should be noted here that participation in scientific research is always associated with the risk of the subject coming to harm. The most frequently mentioned risks include incurring physical, mental, and socioeconomic harm. Research in biomedical sciences often exposes the subject to minor pain, discomfort, or harm caused by medication side effects, and these harms are examples of the physical type of harm. Among the psychological harms, attention should be paid to undesirable changes in the study participants’ thought processes and emotional states, e.g., episodes of depression, confusion or hallucinations after taking the drug, feelings of stress, guilt, and loss of self-esteem. These changes may be temporary, recurrent, or permanent. Potential socioeconomic harms include violations of the right to privacy and intimacy of study participants, job loss, and stigmatization. Moreover, information relating to alcohol or drug abuse, mental illness, illegal activities, and sexual behavior creates areas highly prone to abuse [24].

Applying the principle of beneficence and nonmaleficence, the researcher should assess the significance of the potential benefits and harms and, on this basis, decide on the participation of the subject; bearing in mind the principle according to which a research participant should never be the object of an action or a means to achieve an end. However, as an end in itself, the researcher should assess the moral acceptability of his action.

### 3.2. The Principle of Autonomy

Compliance with the principle of autonomy is based on respecting the patient’s opinion and will. It refers to the freedom of the patient to choose the method of treatment and the need to obtain informed consent from the study participants. Autonomy in bioethics and medicine emphasizes respect for the person and their dignity, including the medic, the patient, and the researcher alike.

On the one hand, hearing loss or deafness require a specialist or researcher, who will provide the patient/study participant with information about the procedure, side effects, risks, benefits, costs necessary to make an autonomous decision; on the other hand, the patient or his or her parents have the opportunity to make decisions based on their beliefs and after consulting specialists or other deaf people. The physician or the researcher in his or her actions is guided by evidence-based medicine and the good of the patient/research participant; nevertheless, he or she is obliged to respect the individual’s will, even if it is not in line with his or her point of view. Involving the patient in the decision-making process is a desirable phenomenon that proves that the principle of autonomy is respected [25,26].

**Figure 2:**
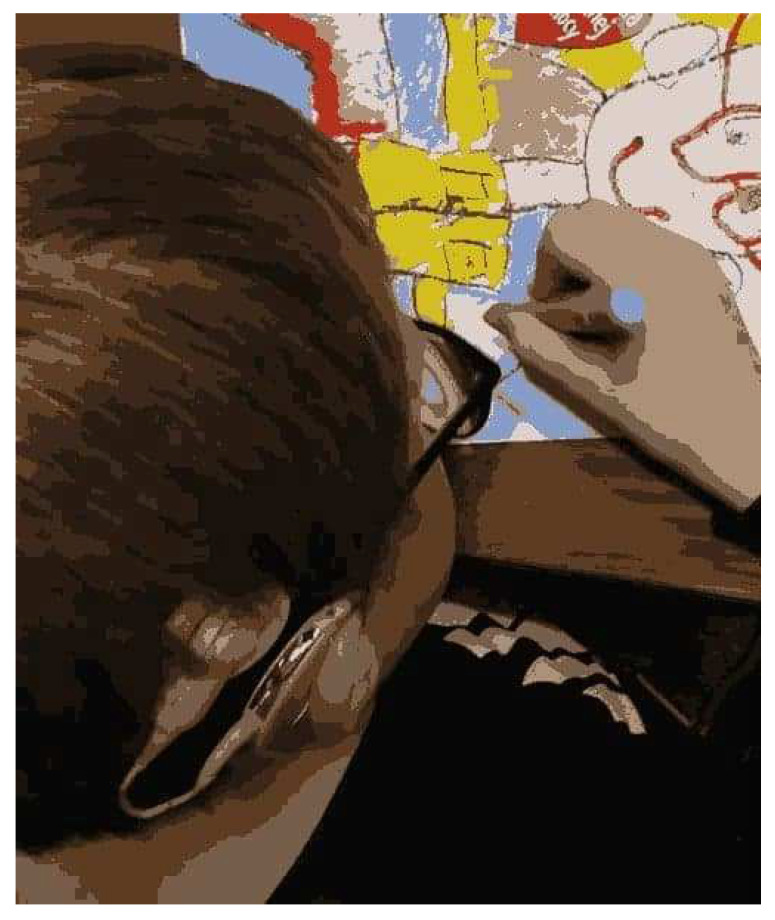
Child with the ear worn cochlear implant processor.

Regarding audiology and the needs of young patients for implant provision, preserving the child’s autonomy is also an essential aspect of medical practice. To maintain the principle of the child’s autonomy, with the literal meaning attributed to it, the intervention decision could be postponed until an older age. However, it should be remembered that for the proper sensorimotor and speech development, it is important to take appropriate action as early as possible [27]. It is also crucial for supporting a child’s psychosocial development (Figure 2).

### 3.3. The Principle of Justice

Another principle of research ethics is the principle of justice. Suppose justice means treating everyone morally and appropriately, including the benefits of participating in the study for all community members. In that case, one should expect equal access for both researchers and, above all, deaf patients to the most modern hearing aids or implants at the best time for a given person. This means that everybody should equally be able to participate in research regardless of, for instance, a gender. Another benefit of participating in research is the application to community members that do not participate in research and to broaden the knowledge and social skills. However, is it so? It seems that both in translational audiology and other fields, especially in modern areas of medicine, the principle of justice functions insufficiently. The application of justice in research should be reflected in social life and the availability of the most appropriate solutions for the patient [28]. Justice in research emphasizes the fundamental principle of “health for all” [27]. This means access to health, regardless of gender, ethnicity, place of birth, political beliefs, religion, economic or social status. Applying the principle of justice in translational audiology could prevent the marginalization of a society. Every researcher in each community would have to provide the best possible equipment implants for the patient, regardless of the patient’s place of residence or economic status.

Consequently, each patient would have the same opportunity to develop communication. It does not always have to be verbal communication; this could involve a sign language interpreter. In the context of the principle of justice, it is essential to inform the patient from the very beginning what solutions they are entitled to regarding the implant and what methods of communication they have at their disposal. Researchers in their daily work should apply the principle of justice. A drawback is that this often leads to the study group being less numerous than if patients were only offered one method of assessment and therapy. Therefore, bearing in mind that clinical trials usually have their origin in medical practice, the ideal solution is if the principle of justice accompanies the researcher from the very beginning of their activity, including conceptualization, practice, and research. However, this is not always the case in everyday life. A body that is responsible for safeguarding, so to speak, the principles of ethics, including justice [29], is the bioethics committee. Therefore, an audiologist who is a researcher has several roles in conducting the study. First of all, he or she should take into account the patient’s well-being and health, as well as the patient’s ethical perspective. Secondly, they must consider factors influencing moral judgment, sensitivity, motivation, courage, and cultural dimensions of ethical practice in audiology [30].

The multifaceted nature of the issues related to deafness also leads to serious moral dilemmas related primarily to reproductive medicine and the treatment of hearing loss. In the case of reproductive medicine, the dilemma may concern the acceptability of donor selection and the selection of an embryo burdened with deafness. Such a situation took place in the USA, where a deaf lesbian couple deliberately created a deaf child. Sharon Duchesneau and Candy McCullough used their sperm donor, a deaf friend with five generations of deafness in his family. Duchesneau and McCullough do not see deafness as a disability. They see being deaf as defining their cultural identity and witness signing as a sophisticated, unique form of communication [31,32,33,34]. The moral dilemma concerns the dispute over the understanding of deafness and the concept of care related to the ethical principles discussed above.

Furthermore, although this problem is not new in ethics, a similar situation occurs when parents do not agree to have a child’s life saved by refusing blood transfusions because of their faith. For this case also, an extremely current problem of rapidly developing research and gene therapies and their applications should be resolved. The question arises whether genetic testing techniques designed to reduce disease and improve the quality of life can be used to deliberate defective embryos. It should be remembered that deafness does mean not only a lack of hearing, which can actually be perceived in a cultural context, but also several comorbidities, including an increased risk of dementia [35]. This dilemma seems to be difficult to resolve from the principles described above. All perspectives boil down to whether it is good that a sick child has been brought into the world. The arguments from one side point to the use of preimplantation genetic diagnostics as incompatible with its objectives. However, it should be remembered that Gauvin, the son of a pair of deaf women, was born thanks to the knowledge in medical genetics. 

## 4. Conclusions

The idea behind translational research in audiology is to improve the quality of medical care for patients with hearing impairment. Although lofty and deserving of the highest recognition, this idea should never be implemented at an individual’s expense in the research procedure. The inalienable dignity of the human being requires researchers and physicians to act for the benefit of the subject, limit the harm, maximize the benefits, and respect the autonomous decisions of the subject. What may serve as signposts indicating the ethical way of conducting translational research in audiology and taking care of patients with hearing impairment are the principles of beneficence, nonmaleficence, autonomy, and justice.

## Data Availability

Not applicable.
